# Leveraging DAGs to improve context-sensitive and abundance-aware tree estimation

**DOI:** 10.1098/rstb.2023.0315

**Published:** 2025-02-20

**Authors:** Will Dumm, Duncan Ralph, William DeWitt, Ashni Vora, Tatsuya Araki, Gabriel D. Victora, Frederick A. Matsen IV

**Affiliations:** ^1^ Computational Biology Program, Fred Hutchinson Cancer Research Center, Seattle, WA 98109-1024, USA; ^2^ Howard Hughes Medical Institute, Chevy Chase, MD 20815, USA; ^3^ Department of Genome Sciences, University of Washington, Seattle, WA 98195-4322, USA; ^4^ Laboratory of Lymphocyte Dynamics, The Rockefeller University, New York, NY 10065, USA; ^5^ Department of Statistics, University of Washington, Seattle, WA 98195-4322, USA

**Keywords:** phylogenetic inference, maximum parsimony, B-cell evolution, somatic hypermutation, affinity maturation

## Abstract

The phylogenetic inference package GCtree uses abundance of sampled sequences to improve the performance of parsimony-based inference, using a branching process model. Our previous work showed that GCtree performs competitively on B-cell receptor data, compared with other similar tools. In this article, we describe recent enhancements to GCtree, including an efficient tree storage data structure that discovers additional diversity of parsimonious trees with negligible additional computational cost. We also describe a suite of new objective functions that can be used to rank these trees, including a Poisson context likelihood function that models sequence evolution in a context-sensitive way. We validate these additions to GCtree with simulated B-cell receptor data, and benchmark performance against other phylogenetic inference tools.

This article is part of the theme issue ‘"A mathematical theory of evolution": phylogenetic models dating back 100 years’.

## 1. Introduction

The adaptive immune system creates antibodies capable of binding to and neutralizing diverse pathogens through an evolutionary process in B cells. B cells produce B-cell receptors (BCRs), which are the membrane-bound form of antibodies. BCRs consist of two protein chains, known as heavy and light, which are encoded by two regions of the B-cell genome. A mutation and selection process, called affinity maturation, takes place in regions of the lymph nodes called germinal centres (GCs). There, B cells rapidly accumulate mutations through a process called somatic hypermutation (SHM), which occurs within the genome regions encoding the two protein chains of their BCRs. Cells whose BCRs demonstrate high affinity for antigen are allowed to proliferate and further diversify, while lineages with lower-affinity BCRs are not. To learn about this process, researchers can extract GCs during affinity maturation, isolate individual B cells and reconstruct evolutionary history [1]. In this intensive sampling of GCs, sampling is dense relative to mutation accumulation. Thus, the same BCR sequence is often observed multiple times, reflecting the relative abundance of the B-cell lineages in the GC.

The phylogenetic inference software GCtree reconstructs the evolutionary history of B-cell affinity maturation, and is designed for the case of intensive sampling of individual GCs. To do so, it uses the PHYLIP program dnapars to build a collection of maximum parsimony (MP) trees relating to the observed BCR sequences [2]. If desired, these observed sequences can be paired with heavy- and light-chain sequences. The resulting set of MP trees often contains significant topological diversity. To decide which tree is most plausible given the data, GCtree computes a branching process likelihood on each, which takes into account the multiplicity of each observed sequence, and encodes the assumption that abundant genotypes are more likely to produce many offspring than scarce genotypes.

This article details improvements to GCtree made possible by the ‘history sDAG,’ a data structure well suited for finding, storing and computing on MP trees [3]. The history subsplit directed acyclic graph (history sDAG) provides efficient storage for phylogenetic histories, including inferred ancestral sequences, through a directed acyclic graph structure that allows equivalent substructures shared by multiple histories to be stored only once. However, this sharing of substructures results in a combinatorial diversity of histories in the history sDAG. That is, the number of unique histories stored in the history sDAG may be greater than the number used to build it. This feature makes the history sDAG especially useful when searching for and storing MP histories because the additional histories found by the history sDAG are guaranteed to be maximally parsimonious if the input histories are also. The history sDAG often expresses orders of magnitude more MP histories than were used to construct it. Fortunately, the data structure also provides efficient means for choosing the optimal history, based on any criterion that can be decomposed as a sum over edges [3].

In this article, we describe improvements to the newest version of GCtree, including a context-based Poisson likelihood, and the use of the history sDAG for storing and efficiently ranking MP trees. We also validate GCtree inference using simulated BCR data, and benchmark performance against other phylogenetic inference tools. We find that the history sDAG significantly improves the ability of GCtree to find trees that are optimal with respect to the likelihoods that GCtree uses for ranking trees, and that this translates to a modest improvement in the accuracy of phylogenetic inference on simulated data.

## 2. Methods

In densely sampled evolutionary data, such as BCR sequences sampled from GCs, it is common to observe some genotypes multiple times. While this abundance information is often ignored by phylogenetic inference algorithms, it reflects the relative abundances of genotypes in the sampled population. GCtree uses this abundance information to compute a branching process likelihood on trees relating to the data, and to choose the most plausible tree from evolutionary scenarios that are equally optimal with respect to MP. The branching process likelihood encodes the intuition that abundant genotypes are more likely to produce many offspring than genotypes that are scarce.

Although implementing this branching process likelihood ranking on MP trees was the original inspiration for GCtree, other ranking criteria can also be used to distinguish between alternative MP trees on data. Indeed, the use of the history sDAG to store MP trees often results in a large increase in diversity in trees to be ranked, both topologically and in variety of ancestral sequence reconstruction. Since the branching process likelihood is sensitive only to topological differences in trees, additional criteria are helpful for choosing between trees that are both maximally parsimonious and equally likely according to the branching process model. The newest version of GCtree includes two new ranking criteria. The first, Poisson context likelihood, is described in the following sections. The other criterion, isotype parsimony, is experimental and described in appendix D. It is provided for comparison by users, but is not yet fully validated.

### (a) Intuitive overview of the history sDAG

**Figure 1.**
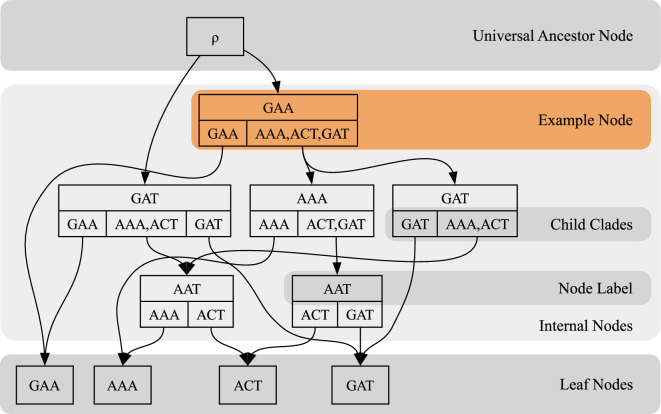
An example history sDAG containing histories on four nucleotide sequences. The highlighted example node v=(ℓ,U) has leaf label ℓ=𝙶𝙰𝙰 and child clades U={{𝙶𝙰𝙰},{𝙰𝙰𝙰,𝙰𝙲𝚃,𝙶𝙰𝚃}}. Its clade union CU⁡(v)={𝙶𝙰𝙰,𝙰𝙰𝙰,𝙰𝙲𝚃,𝙶𝙰𝚃}. Notice that for each edge, the target node’s clade union matches the child clade of the parent node from which the edge descends. A history is a history sDAG with exactly one edge descending from each child clade of each node, and one edge descending from the universal ancestor node.

Here, we provide a brief intuitive overview of the history sDAG; for full details see [3]. The history sDAG compactly represents collections of *histories*, or phylogenetic trees with observed leaf states and inferred ancestral state, as a directed acyclic graph. By including *child clades*, or the sets of taxa reachable beneath a node’s children, as well as inferred ancestral state, as the data of each history node, the history sDAG structure can be defined as a graph union of histories. A node v is a pair (ℓ,U), consisting of the nucleotide sequence ℓ (also referred to as the node’s *label*) and its *child clade set* U, which is a set of sets of leaf labels. Each element of U is a set containing the labels of leaf nodes reachable from one of the children of the node (ℓ,U). Since leaf nodes have no children, their child clade sets are empty. Leaf nodes are required to be labelled uniquely, but multiple internal nodes may share the same label, if they have different child clade sets. The entire set of leaf node labels reachable below a node v will be denoted CU⁡(v) for *clade union*. On leaf nodes, this is the singleton containing the leaf node’s label, and on internal nodes, this is a union of the node’s child clades. In order to keep track of history root nodes, we also introduce a new formal root node ρ, called the *universal ancestor (UA) node*, as a parent of the history’s root. An example of a history sDAG structure is shown in figure 1.

In each history (V,E) with nodes in V as defined above, and E containing pairs of nodes recording directed edges, notice that by definition each edge $\left(v_{p},v_{c}\right)$ must connect its parent node $v_{p}$ to a child node $v_{c}$ such that $\mathrm{CU}(v_{c})$ is one of the child clades of $v_{p}$. Also, each node has exactly one outgoing edge for each of its child clades.

Given a collection of histories $\left\{\left(V_{1}\;,E_{1}),\ldots ,(V_{n}\;,E_{n}\right)\right\}$, a history sDAG can be constructed as their graph union:


$$(V,E)=\left(\overset{n}{\underset{i=1}{⋃}}V_{i}\;,\overset{n}{\underset{i=1}{⋃}}E_{i}\right).$$

Individual histories stored in such a history sDAG (V,E) can be recovered by choosing a single child of the UA node, then for each child clade C of each chosen node v, choosing an additional child $v_{c}$ so that $\mathrm{CU}(v_{c})=C$.

A measurement of phylogenetic optimality can be generalized as a *history weight*, or a function g with values in a totally ordered set of weights W, and taking any phylogenetic history as input. Provided that a history weight can be expressed as the sum of a function f:E→W over all edges $\left(v_{p},v_{c}\right)$ of a history, it is possible to efficiently find the minimum weight of any history in a history sDAG, or trim the history sDAG to express only histories that minimize a weight, using simple dynamic programming algorithms [3].

From such an edge weight function f, we use the notation $g_{f}$ to denote the resulting history weight function:


$$g_{f}(V,E):=\sum_{(v_{p},v_{c})\in E}f(v_{p},v_{c}).$$

Parsimony score is an example of such a history weight decomposable over edges. That is, it is possible to trim a history sDAG to maximally parsimonious histories. Further, although a history sDAG constructed from MP trees may contain more trees than were used to construct it, they are guaranteed to be MP as well. This is a result of theorem 1 in [3].

The details of these algorithms for computing history weights and trimming the history sDAG will be left to the appendix. For the purpose of the present section, it is enough to know that such algorithms exist.

**Figure 2.**
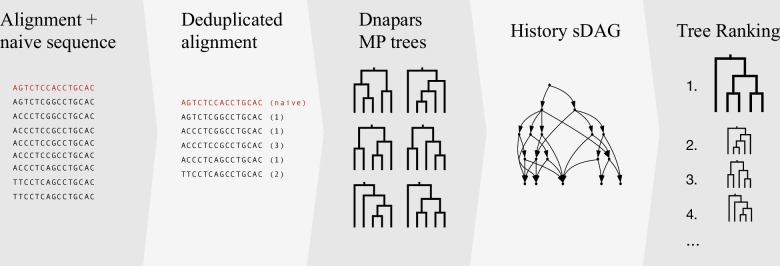
The GCtree inference pipeline updated to include the history sDAG.

GCtree takes as input a collection of maximally parsimonious trees constructed from observed sequences using dnapars, and observed abundances of those samples. Instead of storing these trees in a list, as in previous versions, the newest version of GCtree stores these MP trees in a history sDAG. In addition to often finding many more MP trees to rank, storage in the history sDAG allows more efficient computation of ranking criteria using dynamic programming algorithms. The GCtree inference pipeline, including the history sDAG, is shown in figure 2.

Here, we describe how the history sDAG is incorporated into the new GCtree inference pipeline, and how the branching process likelihood originally used by GCtree to rank MP trees, as well as two new ranking criteria, are computed in the history sDAG.

### (b) Storing dnapars trees in the history sDAG

In the original GCtree inference pipeline, nucleotide ambiguity codes in each dnapars tree were randomly disambiguated, yielding a single maximally parsimonious tree with unambiguous inferred ancestral sequences. Disambiguations of distinct dnapars histories occasionally resulted in the same disambiguated tree, so the number of unique disambiguated histories was in general fewer than the number output by dnapars. The collection of such histories was then used for branching process parameter fitting, and the histories were ranked by likelihood with respect to the fitted model.

In the new GCtree inference pipeline, all histories output by dnapars are used to create a history sDAG. Internal nodes in the history sDAG are then expanded according to the ambiguity codes in their sequence labels. This is equivalent to enumerating all possible maximally parsimonious disambiguations of the dnapars histories. Next, the history sDAG is made *complete* by adding all possible edges to the expanded history sDAG. This is computationally feasible because node pairs can only be joined by an edge when the child node’s clade matches one of the parent node’s child clades. The complete DAG is then trimmed to express only those histories that are maximally parsimonious.

Finally, the history sDAG is collapsed, removing internal edges without mutations, to avoid storing resolutions of multifurcating nodes for which the data provide no support. History sDAG completion and collapsing is described in detail in [3]. This process in general results in a history sDAG structure representing a much greater number of maximally parsimonious histories than are output by dnapars.

### (c) Calculating branching process likelihoods

GCtree makes use of observed sample abundance data to rank maximally parsimonious trees using a branching process likelihood. The model describes the distribution of possible offspring of each individual using a branching probability p and a mutation probability q. Likelihoods according to this branching process model can be factorized over tree edges, and therefore lend themselves to efficient computation in the history sDAG [2].

Before GCtree ranks MP trees according to branching process likelihood, θ=(p,q) is optimized to maximize the marginal likelihood over all MP trees to be ranked. This optimization requires an efficient way to calculate the marginal likelihood over all trees in the history sDAG, as a function of θ. Happily, we can compute this likelihood using a dynamic programming algorithm on the history sDAG directly. This algorithm, as well as the definition of the edge weight function $f_{\theta }$, which yields the branching process log-likelihood of a history given parameters θ, is described in appendix B.

### (d) Additional criteria for sorting maximally parsimonious histories

In practice, the additional histories found by the history sDAG can often make GCtree branching process likelihood rankings degenerate. That is, many different histories may achieve the maximum observed likelihood. To choose the best history in an informed way, we introduce two additional criteria for ranking parsimony histories: Poisson context likelihood, described in this section, and isotype parsimony, which is described in appendix D.

The history sDAG is not necessary for computing these additional criteria, and they could be implemented in GCtree independently of the history sDAG. However, given the large number of MP histories that the history sDAG can find, it is much more efficient to compute these criteria using the history sDAG than on each history independently.

#### (i) Context-sensitive Poisson likelihood

It is well known that during SHM, the rate at which a base mutates is influenced by the bases surrounding it [4,5]. Nearby nucleotides influence both the rate at which a base will mutate, and the distribution of possible target bases. We can incorporate this context sensitivity into a criterion for choosing among trees using a Poisson context likelihood function as follows.

**Figure 3.**
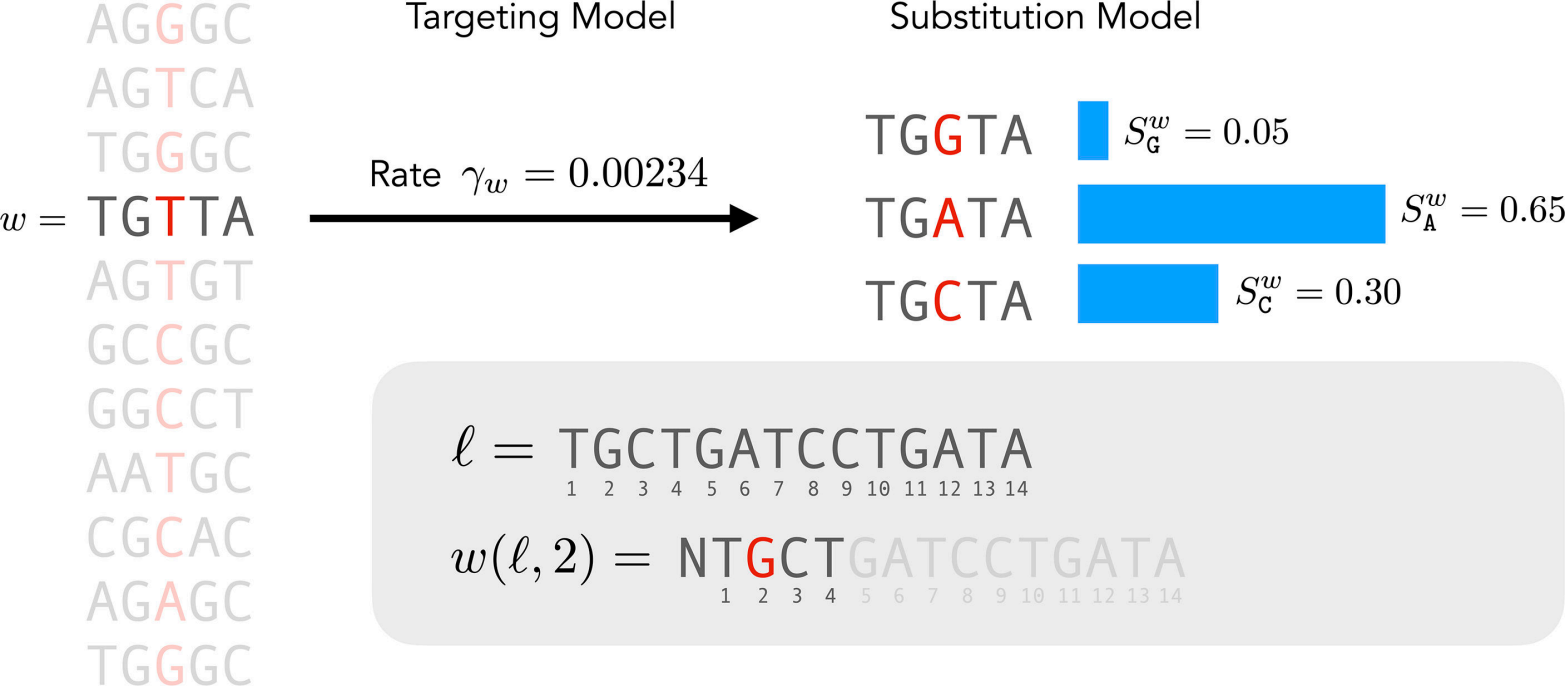
A 5-mer mutability model consists of observed targeting rate and substitution probabilities for the central base in all possible 5-mers. Here we show an example 5-mer w and associated rate $\gamma_{w}$ and substitution probabilities $S_{\text{G}}^{w}$, $S_{\text{A}}^{w}$, and $S_{\text{C}}^{w}$. We also show notation for naming a 5-mer centered at any site in a sequence ℓ. The example 5-mer w(ℓ,2) is shown, padded with a preceding N since site 2 is only one base from the sequence start.

Our goal is to incorporate the context sensitivity of mutation rates using the following type of model. Let M be the set of 1024 possible nucleotide 5-mers. That is, each element of M is a sequence containing five bases in {A,G,C,T}. A mutability model consists of two parts. The first is a targeting model describing the normalized rates $\gamma_{w}$ at which a mutation will occur at the central base of each 5-mer $w=(w_{1},\ldots ,w_{5})\in M$. The second is a substitution model consisting of, for each 5-mer in w∈M, and each of the three bases *B* that the central base $w_{3}$ of w can mutate to, a probability $S_{B}^{w}$ that $w_{3}$ will mutate to B given the 5-mer context w. Such a model is depicted in figure 3.

If we assume no mutations in the flanks of the 5-mer and no multiple mutations at a site, we can interpret this model to mean that the central base $w_{3}$ in the 5-mer w mutates to a new base B in time τ with probability $1-e^{-\gamma_{w}S_{B}^{w}\tau }$ [6].

Complete 5-mer context may not be available for the first two or last two sites in a sequence. To handle this, we treat the missing context as unknown, and compute the mean mutabilities and targeting probabilities over all compatible 5-mers. For example, in figure 3, the 5-mer w(ℓ,2) contains a single ambiguous N base, so the mutabilities and targeting probabilities for the central base are the means of the values over all four 5-mers that can be made by replacing that N with a base.

This context-based Poisson tree likelihood decomposes over the edges of the history. Let ℓ and $\ell^{\prime }$ be the parent and child sequences of a branch, respectively, and let w(ℓ,j) be the 5-mer centred at site j of ℓ. Also define the Poisson rate vector $\lambda (\ell ,\ell^{\prime }{)}_{j}=\gamma_{w(\ell ,j)}S_{\ell_{j}^{\prime }}^{w(\ell ,j)}$. Finally, define $n(\ell ,\ell^{\prime })$ to be the number of sites that differ between the aligned parent and child sequences ℓ and $\ell^{\prime }$. We can then define the following Poisson log-likelihood for a branch whose parent sequence is ℓ and whose child sequence is $\ell^{\prime }$. Recall that a pair (ℓ,U) is a vertex in the history sDAG:


$$f_{C}\left(\left(\ell ,U),(\ell^{\prime },U^{\prime }\right)\right)=\left(\sum_{j|\ell_{j}\neq \ell_{j}^{\prime }}\log(\lambda (\ell ,\ell^{\prime }{)}_{j})\right)+\log(\hat{t})n(\ell ,\ell^{\prime })-n(\ell ,\ell^{\prime }),$$

where we have taken the branch length to be the maximum likelihood estimate, namely,


$$\begin{array}{c} \hat{t}=\frac{n(\ell ,\ell^{\prime })}{\sum_{j}\gamma_{w(\ell ,j)}}. \end{array}$$

Here, we assume that only one mutation may occur at each site on a branch. The derivation of this likelihood is provided in appendix C.

The log-likelihood of an entire evolutionary history is the sum of all its branch log-likelihoods as defined above. Notice that since the branch log-likelihood is 0 if there are no mutations between ℓ and $\ell^{\prime }$, this history likelihood is not sensitive to collapsing of edges without mutations.

All trimming and weight annotation lemmas in appendix A apply to this Poisson context log-likelihood $f_{C}$, since it is a real-valued function on history edges.

#### (ii) Combining weight functions

The history sDAG can be trimmed to express only those histories that minimize a lexicographical ordering of history weights, since one can trim the history sDAG iteratively with respect to each weight function in descending order of importance. The default behaviour of GCtree, if provided with isotype data and a mutation model, is to rank MP trees lexicographically, first maximizing branching process likelihood, then minimizing isotype parsimony, and finally maximizing Poisson context likelihood.

For a sequence of edge weight functions $f_{1},f_{2},\ldots ,f_{n}$ that all take values in ℝ, with the standard notion of addition, history sDAG trimming and weight computations can also be performed using any linear combination of the corresponding history weights. In particular, notice that for a linear combination $f^{\prime }=a_{1}f_{1}+a_{2}f_{2}+\ldots +a_{n}f_{n}$, one has


$$\begin{array}{c} g_{f^{\prime }}=a_{1}g_{f_{1}}+\ldots +a_{n}g_{f_{n}}. \end{array}$$

This is true because $g_{f}^{\prime }$ is a sum of the edge weight function $f^{\prime }$ applied to all edges in a history.

Optionally, GCtree can be provided with coefficients defining a history weight, which is a linear combination of branching process likelihood, isotype parsimony, and Poisson context likelihood. This allows inference to choose a tree that is slightly suboptimal with respect to one criterion if it allows another criterion’s value to be significantly more optimal.

### (e) Simulation of B-cell evolution

We simulate the evolution of BCR sequences using a revised version of bcr-phylo [7] under a model that simulates both the mutation process and a model selection process. bcr-phylo is a birth–death simulator with context-sensitive mutation according to an S5F model, and fitness-dependent branching. The S5F model used is based on human SHM data, and distributed as part of the SHazaM package [4,8].

Our modifications to bcr-phylo were intended both to make the simulation more generally realistic, as well as to more closely mimic sequences from a GC sequencing experiment extending the work of [1]. We simulate the evolution of heavy- and light-chain BCR sequences together, with amino acid Hamming distance to a single ‘target’ sequence representing a hypothetical ideal antibody. Simulation begins with 100 initial individuals sharing a common naive sequence 10 amino acid mutations away from this target sequence, with affinity (and consequently fitness, or expected number of offspring) increasing for sequences that mutate toward the target. Sequence length is approximately 700, but varies depending on naive sequence choice. Cells initially multiply without restriction, but as their total number nears the carrying capacity of 1000, this slows as they compete for a limited amount of available antigen. Once sequences are closer to the target sequence than a distance of 2, further distance decreases do not change affinity. Out of all the positions in the combined heavy and light sequences, we select 60 at random to use as ‘paratope’ positions, i.e. that affect the target distance calculation. A further 100 positions are similarly selected as ‘structural’ positions that are permitted only synonymous mutations (and do not affect the target distance), while the remaining positions mutate freely under the constraints of the S5F model, and also do not affect target distance. This partitioning into paratope, structural and unrestricted positions has the important effect of focusing mutation onto a relatively small number of positions, as we see in the data. The weight of each ‘paratope’ position in the sequence in the distance calculation is chosen uniformly randomly from the interval [0,1]. This represents the empirical fact that a mutation can have a very large or very small effect on affinity, depending on its position. After the desired number of generations (between 15 and 50), we sample 70 cells from the tree. We rank the cells in terms of affinity at the final time point and take the top 70 as our sampled set of sequences. This sampling scheme gave an abundance distribution matching real data, which was a primary goal of our simulation. We repeat this procedure for 210 different trees, and split these into three replicates of 70 each in order to calculate uncertainties as standard errors. Simulation parameters are summarized in table 1, alongside the simulation parameters for the previous version.

**Table 1.** Simulation parameter values for the current paper compared with those from [7]. All parameters not listed are left at their default values. In the current paper, we sample 70 sequences from each simulated germinal centre (GC), all from the final generation. This final generation value, however, varies across the different samples from 15 to 50. In [7], on the other hand, for all GCs, 30 sequences were sampled at the final generation 45 as well as at the intermediate generations 15 and 30. Parameters with ‘n.a.‘ were not present in the simulation version used in [7].

parameter	current value	value in [7]
no. target sequences	1	100
target distance	10	5
target distance weights	uniform random	n.a.
minimum target distance	2	n.a.
no. total sampled sequences	70	90
no. sampled sequences per generation	70	30
sampled generations	15−50	15, 30, 45
paratope positions	*n* = 60	n.a.
structural positions	*n* = 100	n.a.
leaf sampling scheme	high affinity	uniform random
carrying capacity	1000	1000
no. initial sequences	100	n.a.

We then use the paired heavy- and light-chain sequences from sampled lineages to infer histories using GCtree and other methods, and compare with the true evolutionary relationships. Inferential accuracy of the methods is evaluated on simulations run for between 15 and 50 generations.

### (f) Inference methods

#### (i) GCtree in multiple configurations

In order to evaluate the performance of the GCtree ranking criteria, both separately and together, in choosing the most realistic MP tree, we run GCtree inference in three different configurations.

First, for a comparison with the baseline performance of branching process likelihood alone, we use GCtree v. 3.3.0, which does not include the new ranking criteria or history sDAG features described in this article. Inference is based only on branching process likelihood, without using any other ranking criteria. This configuration is referred to as 'GCtree (no DAG)'.

Second, to evaluate the performance of the GCtree Poisson context likelihood alone, we provide the newest version of GCtree (v. 4.3.0) with the S5F model used in sequence simulation, and ignore all other ranking criteria. This configuration is referred to as 'GCtree (context)'.

Finally, to evaluate the performance of the complete updated GCtree inference method, we provide the inference pipeline with the S5F model used in sequence simulation, but allow branching process likelihood to be used as well. Ranking of MP trees is performed lexicographically, first maximizing branching process likelihood, and then maximizing Poisson context likelihood. This is referred to as 'GCtree'.

None of these GCtree configurations evaluates the performance of isotype parsimony as a ranking criterion.

#### (ii) IgPhyML

IgPhyML is another maximum-likelihood tool, designed specifically for phylogenetic inference on paired heavy- and light-chain BCR sequences [9,10]. IgPhyML uses the HLP19 model, which accounts for known features of SHM such as the ratios of non-synonymous to synonymous mutations and transitions to transversions, as well as the variance of site mutability with nucleotide context. We ran IgPhyML v. 2.0.0 in standalone mode, with the –ASR option to perform ancestral sequence reconstruction, and the threshold for ambiguous bases in ancestral sequences (–ASRc) set to 0 to avoid ambiguous bases. Because of difficulties in specifying the heavy- and light-chain information in standalone mode, we passed in only a sequence file, such that IgPhyML estimated only a single ω and branch rate for both heavy and light chains. A recent publication shows little difference in topological accuracy when using a single partition for both chains [11].

#### (iii) RAxML

RAxML is a tool for maximum-likelihood phylogenetic inference, optimized for large alignments on many sites [12]. Unlike GCtree, which first optimizes trees with respect to parsimony, and uses likelihood to decide between alternative parsimony trees, RAxML optimizes a tree to maximize likelihood directly. We used RAxML-NG v. 1.2.1 with the GTR+G model and the –ancestral option to perform ancestral state reconstruction at all internal nodes.

#### (iv) IQ-TREE

Finally, IQ-TREE is another widely used package for general maximum-likelihood tree reconstruction. We used v. 1.6.12 with the -asr flag for ancestral sequence reconstruction, but otherwise used default parameters.

### (g) Comparing inferred and simulated trees

In order to explore the performance of GCtree at reconstructing histories of B-cell evolution, we infer histories from simulated data using GCtree and a variety of other phylogenetic tools. We then compare the reconstructed histories with the true, simulated histories to evaluate the relative accuracy of inference between the methods.

To compare inferred trees with true simulated phylogenies, we use the *most recent common ancestor (MRCA) distance*. For each pair of taxa, we calculate the Hamming distance between the true and inferred MRCA node sequences, then divide by the mean Hamming distance between each of the taxa and the true MRCA node. This is then summed over all taxa pairs. Notice that since the choice of MRCA node for two taxa is unique on any tree, this distance is well defined even when one or both of the trees being compared contains multifurcations.

Unlike metrics such as Robinson–Foulds (RF) distance that consider only tree shape, MRCA considers the correctness of ancestral sequence reconstruction, which is often an important part of phylogenetic analysis of BCR data. We also considered the COAR metric from [7]; however, we found that its score was often dominated by cases where its alignment algorithm incorrectly aligned two highly divergent sequences when the true and inferred lineages had a significantly different number of members.

## 3. Results

### (a) The history sDAG enables many more trees to be ranked and does so efficiently

Because the history sDAG finds more MP trees, the new version of GCtree will consider for ranking a superset of the MP trees considered for ranking by previous versions of the pipeline. As a result, the optimal MP tree chosen by GCtree will rank as high as that which would have been chosen by previous versions, with respect to the ranking criteria used. With our simulated data, we can quantify the extent to which we find additional trees to rank, and the extent to which this leads to an improvement in the ranking criteria.

**Figure 4.**
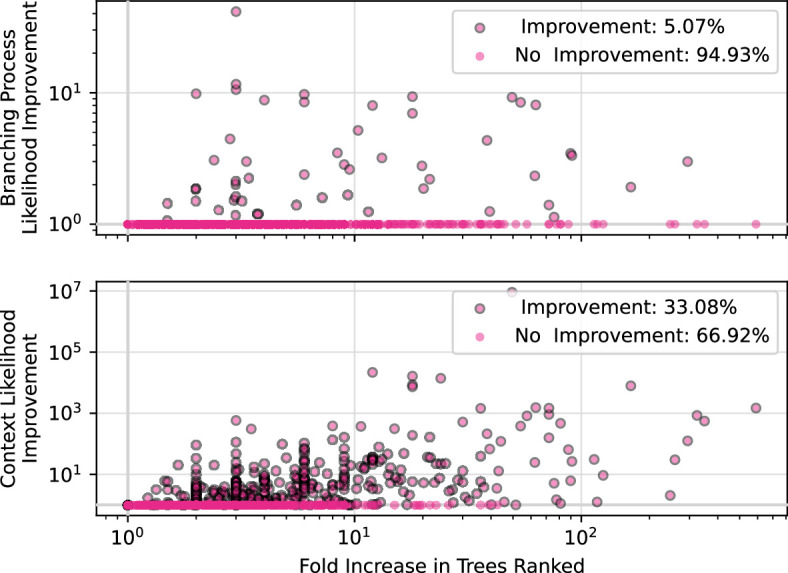
For each simulated dataset, the factor by which the history sDAG increases the number of trees available for ranking, and the ratio of the best branching process and Poisson context likelihood of the best tree in the history sDAG to those of the best tree found by dnapars. Bold grid lines show a ratio of 1, or no improvement in the history sDAG compared with trees from dnapars.

Indeed, we find that the history sDAG finds new MP trees that can score better with respect to ranking criteria, on an assortment of realistic simulated datasets (figure 4).

**Figure 5.**
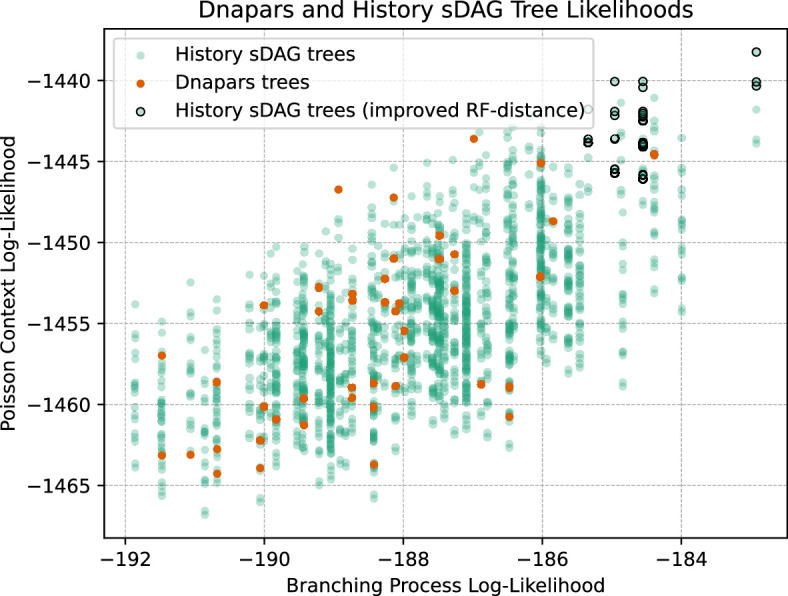
For a single simulated dataset, we show the Poisson and branching process likelihood of each tree inferred by dnapars and the history sDAG. Black-bordered points show trees found by the history sDAG that are more similar to the true simulated tree than any found by dnapars, with respect to normalized Robinson–Foulds (RF) distance. The trees found by the history sDAG can significantly increase ranking likelihoods and correctness.

To illustrate how the history sDAG improves these likelihoods, we show for a single example simulated dataset the branching process likelihood and Poisson context likelihood for each tree found by dnapars, and each additional tree found by the history sDAG (figure 5). In addition, the figure shows trees found by the history sDAG that are closer to the true simulated tree (with respect to normalized RF distance) than any tree found by dnapars. Trees found by the history sDAG have significantly better likelihoods than the best trees found by dnapars. In this example, the better likelihoods also correspond to improved tree correctness compared with the best tree found by dnapars.

In addition to finding in some cases millions of new maximally parsimonious histories, the history sDAG allows efficient computation on these histories using dynamic programming, as described in appendix A. The dynamic programming algorithm for finding the minimum history weight in a history sDAG (V,E) has time complexity O(|E|). In the worst case, when the histories have no edges in common, this is no better than finding the minimum history weight by computing the weight of each history independently. However, when a history sDAG finds new histories by allowing new combinations of substructures, edges are necessarily shared between those histories. That is, we can guarantee that trimming and minimum weight computations on the histories in the history sDAG will be no slower than the equivalent serial computation on the individual histories used to build the history sDAG. In practice, the histories represented by the history sDAG are often highly similar, and finding the optimal history in the history sDAG via dynamic programming represents a very significant improvement in time complexity.

Even on datasets for which the history sDAG found millions of maximally parsimonious histories, GCtree parameter inference takes less than a minute, and searching for the optimal tree takes seconds. Parameter fitting on millions of histories would have taken many hours before the addition of the history sDAG.

Finally, the history sDAG allows very compact storage of collections of similar, maximally parsimonious histories. For example, for the datasets summarized in figure 4, the largest serialized history sDAG is 3 MB, and most are under 500 kB, despite containing all the information for up to $10^{26}$ unique histories. By comparison, the serialized data structure previously used by GCtree to store histories would typically have consumed more than 50 kB of storage per history, or 50 GB per million histories. This compression mirrors that seen in other areas with similar data structures [13].

### (b) Additional criteria modestly improve inference accuracy

**Figure 6.**
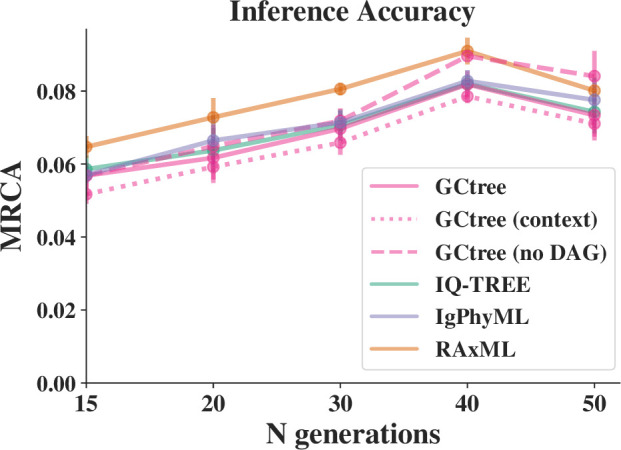
Inference performance of the phylogenetic tools as a function of the number of simulated generations. The most recent common ancestor (MRCA) metric measures the distance between the true and inferred MRCA nodes for each pair of taxa (see text).

The two updated GCtree methods perform slightly better than the other tested methods throughout most of the tested range (figure 6), while the performance of (original) GCtree (no DAG) degrades slightly at higher mutation levels. This indicates that the additions made to GCtree, in aggregate, are effective at improving inferential accuracy. IQ-TREE and IgPhyML perform similarly, while RAxML performs somewhat worse. The differences among the different methods are, however, not particularly large: the methods all correctly infer between about 93 and 95% of the differences between each pair of taxa and their common ancestor sequence at the middle observation times (an observation time of 30 corresponds to about 1.5% nucleotide SHM).

GCtree (context) completely ignores abundance information, and only uses an S5F mutability model to choose between MP trees. It may not be surprising that this GCtree configuration performs well on the simulated data, since neutral mutations are simulated using the same S5F mutability model (although these sequences are also subject to a process of natural selection). However, in settings where an appropriate experimentally determined S5F model is available for the data, this shows that exclusively using context for GCtree inference may be effective, especially when sequences are rarely sampled multiple times and therefore abundance information is uninformative.

While IQ-TREE performs slightly worse than GCtree on these simulations, it is important to note that it is also notably faster in most cases, especially when the diversity of MP trees on the data is large.

We found minimal differences in the reconstruction inference in terms of the RF distance (results not shown).

## 4. Discussion

In this article, we describe enhancements to the phylogenetic inference package GCtree, and show that they modestly improve performance on simulated BCR data. The first enhancement is the use of the history sDAG data structure to store MP trees, which often results in a greatly expanded diversity of trees for ranking purposes (figure 4). We also provide new criteria for ranking MP trees, including Poisson context likelihood, and show that when used together with branching process likelihood these criteria modestly improve the correctness of the inferred tree under simulation, relative to branching process likelihood alone. However, we observe that the accuracy of GCtree is very similar to all other inference tools benchmarked (figure 6).

One surprising result is that the GCtree (context) configuration, which uses only the Poisson context likelihood, performs slightly better on the simulated data than GCtree, which uses both branching process likelihood and Poisson context likelihood. This is likely because the S5F model used in the simulations is identical to the S5F model used to compute the Poisson context likelihood. The GCtree configuration does consider Poisson context likelihood when ranking trees, but it first chooses trees that maximize branching-process likelihood. Figure 6 shows that context likelihood alone performs somewhat better on the simulated data than using branching process likelihood alone, so ranking first to maximize branching process likelihood restricts the set of trees considered for further ranking by context likelihood, reducing its effectiveness. However, on real data, the S5F model may not perfectly match the true mutabilities and substitution probabilities, and the branching process likelihood may be more informative than the Poisson context likelihood.

Unlike the other tools benchmarked, the primary phylogenetic criterion used by GCtree is MP. The ranking criteria described in this article are only used to choose the most plausible tree among the MP trees found by dnapars on the data. In contrast, other methods use more sophisticated phylogenetic criteria directly while searching tree space for plausible trees. However, GCtree’s approach is competitive on BCR data, as figure 6 shows. This is likely because MP is a close approximation to maximum-likelihood methods on densely sampled data, such as BCR sequences [14]. MP is also a simple criterion, with respect to which the most plausible regions of tree space can be discovered and summarized relatively quickly. GCtree leaves all exploration of tree space to dnapars. Optimizing tree structure directly according to the GCtree ranking criteria would be a significantly more complicated task than what GCtree currently does.

Previous work showed that the original version of GCtree performed significantly better than all other methods tested here [2,7]. However, on the simulations in this article, we see that while GCtree using all ranking criteria still slightly outperforms other methods, GCtree with analogous inference options (GCtree (no DAG)) to the old version actually performs slightly worse than other methods. This disagreement is likely due to differences in the simulation setup used in these validations, which are summarized in SimuParams. As described above, we have worked to improve the realism of the simulations for the specific case of a densely sampled GC. All three validations agree that GCtree accuracy is very similar to that of other inference methods.

## Data Availability

The version of GCtree (v. 4.3.0) benchmarked in this article can be installed using pip and Conda and is on Github [2]. The code to produce simulations, validate inference methods, and produce plots is available at [15]. In preparing our analysis and plots, we used the Python packages ETE3 [16], Polars [17] and Matplotlib [18].

## Appendix A. Histories and the history sDAG

See table 2.

**Table 2.** Notation used in the text.

***labels***		
	ℓ	a node label, such as a nucleotide sequence
	ρ	the universal ancestor node label
	C	a clade, i.e. a subset of leaf labels
	U	a set of disjoint clades
***histories***		
	t	a history
	v	a node in a history or history sDAG
	f	an edge weight function on pairs of history or history sDAG nodes
	$f_{\theta }$	an edge weight function for branching process likelihood with parameters θ
	$f_{I}$	an edge weight function for isotype parsimony
	$f_{C}$	an edge weight function for Poisson context likelihood
	$g_{f}$	a weight function on histories, summing f over all edges
	CU⁡(v)	the clade union of a node v
***history sDAGs***		
	V	a set of history sDAG or history nodes
	E	a set of history sDAG or history edges constructed from histories T
	Ch⁡(v)	children of a node v in a history or history sDAG

### Accumulating history weights by dynamic programming

In previous work, we described dynamic programming algorithms for efficiently computing the minimum weight of any history in a history sDAG, and for trimming the history sDAG to contain only histories of minimum weight. Definition 15 and lemma 11 in [3] describe this trimming algorithm. In this appendix, as in the cited work describing the trimming algorithm, we will refer to a subgraph of a history, including exactly all the nodes and edges below and including a chosen subhistory root node, as a *subhistory*. The trimming algorithm involves first computing, for each node in the history sDAG, the minimum weight of any subhistory rooted at that node. Then, we can visit each node in the history sDAG and eliminate each child edge that does not participate in a minimum-weight subhistory. This step can leave nodes orphaned in the history sDAG, so we must then remove any nodes that are no longer reachable from the UA node. As the cited lemma shows, the resulting history sDAG is guaranteed to contain exactly the minimum-weight histories from the original history sDAG.

Here, we describe a similar dynamic programming algorithm to summarize the weights of all histories contained in the history sDAG. Although the worst-case performance of this algorithm is equivalent to individually computing the weight of each history in the history sDAG, we can expect it to be efficient in the common setting when many histories share the same weight.

Recall that each history in a history sDAG has exactly one descendant edge from each node–clade pair, and contains the UA node and each leaf node. Also, given a node (ℓ,U) in a history sDAG (V,E), and a clade C∈U, the shorthand

$$\mathrm{Ch}\left((\ell ,U),C\right):=\left\{(\ell^{\prime },U^{\prime })\in V\;|\;\left(\left(\ell ,U),(\ell^{\prime },U^{\prime }\right)\right)\in E,\;\mathrm{CU}(\ell^{\prime },U^{\prime })=C\right\}$$

will denote the set of nodes that are children of the node–clade pair ((ℓ,U),C).

We will continue to employ standard set-builder notation, but replace curly brackets with square brackets to denote an unordered sequence. In practice, we can implement this algorithm efficiently by replacing these unordered sequences with multisets, relying on the fact that identical weights are often encountered on many unique subhistories.

The following lemma allows efficient accumulation of weights of histories in the history sDAG by calculating and storing weights of subhistories rooted on all nodes in the history sDAG, starting at the leaves.

In preparation for lemma A.1, let f be an edge-weight function from pairs of history sDAG nodes to W, a weight set, endowed with (and closed under) addition, with 0∈W a formal additive identity element.

The weight of a history (or subhistory) will be $g_{f}$, a sum of f evaluated on all edges of the history.

We will recursively define a function G from history sDAG nodes to finite sequences of weights: For any leaf node v, let G(v):=[0], the sequence containing only the additive identity of W.

Next, for a non-leaf node v=(ℓ,U), and a particular clade C∈U, let

$$\begin{array}{c} G_{C}(v):=\left[w+f(v,v_{c})\;|\;v_{c}\in \mathrm{Ch}(v,C),w\in G(v_{c})\right]. \end{array}$$

That is, $G_{C}(v)$ contains all weights possible on subtrees below the clade C, including the weight of the edge connecting them to v.

Finally, for any non-leaf node v=(ℓ,U) with n child clades, let

$$\begin{array}{c} G(v):=\left[\sum_{i=1}^{n}w_{i}\;|\;(w_{i}{)}_{i=1}^{n}\in \prod_{C\in U}G(v,C)\right]. \end{array}$$

**Lemma A.1**. *The sequence* G(v) *lists the weight of each subhistory rooted at* v*, and therefore* G(ρ) *lists the weights of all histories in the history sDAG.*

Note that the product in the definition of G(v) is the Cartesian product on the n finite sequences G(v,C) for each C∈U.

*Proof*. The proof is by induction. Below any leaf node, there is just one possible subhistory containing no edges, so the sum of f over the edges of such a tree is by convention 0. That is, G(ℓ,∅)=[0] correctly lists the weights of subhistories below leaf nodes.

Now assume that for v=(ℓ,U), a history sDAG node, the function $G(\ell_{c},U_{c})$ correctly lists the weights of possible subhistories below $\left(\ell_{c},U_{c}\right)$ for each child node $\left(\ell_{c},U_{c}\right)$ of (ℓ,U). Then, notice that a choice of subhistory rooted at (ℓ,U) is a choice of target node for each clade C∈U, and a choice of subhistory below each chosen target node. Notice that such possible choices are in bijection with the elements of G(ℓ,U), and that for each subhistory constructed in this way, its weight is the sum of each below-target subhistory weight, plus the weight of its parent edge. These are exactly the elements of G(v), given that the conclusion is true for child nodes of v.∎

## Appendix B. Branching process likelihoods in the history sDAG

The branching process model consists of two parameters θ=(p,q), and defines a distribution on *fully collapsed* trees with sequence labels at each node [2]. Fully collapsed means that no edge connects two nodes with the same sequence label.

The history sDAG cannot accommodate fully collapsed trees, since pendant edges cannot be eliminated, even when they lack mutations. However, the history sDAG can be collapsed, in the sense that all non-pendant edges without mutations are collapsed [3]. Fortunately, histories in the collapsed history sDAG constructed with sequence data as labels are in bijection with the fully collapsed trees considered by GCtree’s branching process model. This makes computation of branching process likelihood in the history sDAG convenient.

Branching process model likelihoods can be factorized as $\prod_{i}h_{\theta }(c_{i},m_{i})$, where the product is over nodes in a fully collapsed tree, $c_{i}$ and $m_{i}$ are the observed abundance and number of descendants of node i, respectively, and h is an easily computable function with values in the unit interval [2].

This decomposition over edges in a history makes computing the likelihood trivial in the history sDAG, given values for p and q. However, since p and q must be optimized to maximize the marginal likelihood of all MP trees, we additionally need to be able to compute the marginal likelihood over all histories in the history sDAG. Since the factorized likelihood depends only on the abundance and number of descendants of each node in a history, a (unordered) list of abundances and child counts of each node in a history is sufficient to calculate the likelihood of a fully collapsed tree under this model. By summarizing the sets of node abundances and child counts in all histories in the history sDAG, we can compute the marginal likelihood of histories in the history sDAG as a function of branching process parameters.

To do this, we can use lemma A.1 to keep track of history weights in a weight set W, consisting of unordered sequences of non-negative integer pairs (c,m), where c is a node abundance, and m is the number of mutant children below that node. A history’s weight in W is the unordered sequence of pairs describing the abundance and number of mutant children of each of its nodes. We will use square brackets to denote these unordered sequences. Addition on this weight set is sequence concatenation, and the formal identity 0∈W is the empty sequence []. Since we will use this weight set only for computing the marginal likelihood over all histories in the history sDAG, and not for trimming the sDAG, it need not be endowed with a total ordering.

Let $a:S\to {\mathbb{N}}_{0}$ be a map specifying the observed abundance of each label, and define the weight of an edge from a parent to a child as

$$\begin{array}{r} f_{L}\left(\left(\ell_{p},U_{p}),(\ell_{c},U_{c}\right)\right)=\left\{ \begin{array}{ll} \left[\;\right] & U_{c}=\emptyset ,\ell_{p}=\ell_{c}\\ \left[\left(a(\ell_{c}),|U_{c}|-1\right)\right] & \left\{\ell_{c}\right\}\in U_{c}\\ \left[\left(a(\ell_{c}),|U_{c}|\right)\right] & \text{ otherwise}. \end{array}\right. \end{array}$$

Notice that $f_{L}$ outputs sequences that are either empty or contain a single such integer pair consisting of the abundance and number of mutant clades of the target node. In order to accommodate the fact that pendant edges may not be collapsed, $f_{L}$ returns an empty sequence when evaluated on pendant edges whose parent and child genotypes match. A node that is the parent of such an uncollapsed edge has one non-mutant child node, and therefore the number of mutant children is one fewer than the cardinality of its child clade set, as expressed in the second case in the definition of $f_{L}$.

The sum of $f_{L}$ applied to all edges in a collapsed history gives a list of (c,m) pairs for all nodes in the corresponding fully collapsed tree.

Define a function $L_{\theta }:W\to [0,1]$, where L(0)=1, and

$$L\left([(c_{i},m_{i}){]}_{i=1}^{n}\right)=\prod_{i=1}^{n}h_{\theta }(c_{i},m_{i}).$$

Using this function to compute the branching process likelihood of each tree in the history sDAG given parameters θ, we can use lemma A.1 and the edge weight function described above to accumulate weights of all trees in the history sDAG. We can then compute the marginal likelihood, and optimize θ as in [2]. Distinct histories in the history sDAG often contain identical node abundances and child counts, so this process can in practice be done quite efficiently by caching computations from previously observed likelihood computations.

Once the branching process parameters θ are fitted, we can use the real-valued edge weight function $f_{\theta }=\ln\;\circ L_{\theta }\circ f_{L}$ to compute and maximize the log-likelihood of histories in the history sDAG.

## Appendix C. Derivation of Poisson context likelihood

We want to formulate a Poisson likelihood that uses site and targeting base-specific rates. This will describe the likelihood of one sequence mutating to another, along a branch with length t.

Suppose we have a rate $\lambda_{j,B}(X;\theta )$ for every site j in a sequence, and alternative base *B*. We then have a total $\lambda_{j}(X;\theta )=\sum_{B}\lambda_{j,B}(X;\theta )$ where the sum is over all alternative bases.

Let $n_{j}$ be a 0/1 indicator of mutation at site j, and let $n_{j,B}$ be a 0/1 indicator of mutation at site j to base *B*.

For modelling, we assume that $n_{j}=1$ means that there is exactly one substitution that has happened, and that no more mutations are possible. This is different from saying that there is at least one mutation. In statistical language, this means that our 0/1-valued mutation indicator comes from a full, non-censored Poisson distribution rather than using a censored Poisson likelihood.

Making that assumption, the likelihood is

$$\prod_{j,B}\frac{{\left(t\lambda_{j,B}\right)}^{n_{j,B}}e^{-t\lambda_{j,B}}}{n_{j,B}!}=\left[\prod_{j}{\lambda_{j,B}}^{n_{j,B}}\right]t^{\sum_{j,B}n_{j,B}}e^{-t\sum_{j,B}\lambda_{j,B}}.$$

(Because we are making the 0−1 assumption, we can drop the $n_{j,B}!$.) What is nice about this is that it splits into two components: the first part actually depends on the mutations that happened, but does not have to do with time, and the second has to do with time and does not have to do with the mutations that happened. This follows from Poisson thinning/superposition.

Since $\sum_{j,B}n_{j,B}=\sum_{j}n_{j}$, and $\sum_{j,B}\lambda_{j,B}=\sum_{j}\lambda_{j}$, the above equation can be simplified to the following log-likelihood:

$$\left(\sum_{j,B}n_{j,B}\;\log\;(\lambda_{j,B})\right)+\left(\sum_{j}n_{j}\right)\log(t)-t\sum_{j}\lambda_{j}.$$

Notice that the first term does not depend on t. Fixing the $\lambda_{j}$’s, the following value of t maximizes the likelihood:

$$\hat{t}=\frac{\sum_{j}n_{j}}{\sum_{j}\lambda_{j}}.$$

Finally, by substituting $\hat{t}$ for t, we get the following maximized branch likelihood:

(C 1)
$$\begin{array}{ccc} & \left(\sum_{j,B}n_{j,B}\log(\lambda_{j,B})\right)+\left(\sum_{j}n_{j}\right)\;\log\left(\frac{\sum_{j}n_{j}}{\sum_{j}\lambda_{j}}\right)-\sum_{j}n_{j}. & \end{array}$$

To compute this likelihood using the experimentally determined mutabilities and targeting probabilities in an S5F model, let ℓ and $\ell^{\prime }$ be the parent and child sequences of a branch, respectively, and let w(ℓ,j) be the 5-mer centred at site j of ℓ. Also, define the Poisson rate vector $\lambda (\ell ,\ell^{\prime }{)}_{j}=\gamma_{w(\ell ,j)}S_{\ell_{j}^{\prime }}^{w(\ell ,j)}$. Finally, define $n(\ell ,\ell^{\prime })$ to be the number of sites hat differ between the aligned parent and child sequences ℓ and $\ell^{\prime }$. We can then modify equation (C 1) using rates from the S5F model, to compute the Poisson log-likelihood for a branch whose parent sequence is ℓ and whose child sequence is $\ell^{\prime }$:

$$f_{C}\left(\left(\ell ,U),(\ell^{\prime },U^{\prime }\right)\right)=\left(\sum_{j|\ell_{j}\neq \ell_{j}^{\prime }}\log(\lambda (\ell ,\ell^{\prime }{)}_{j})\right)+\log(\hat{t})n(\ell ,\ell^{\prime })-n(\ell ,\ell^{\prime }),$$

where the maximum likelihood branch length is

$$\begin{array}{c} \hat{t}=\frac{n(\ell ,\ell^{\prime })}{\sum_{j}\gamma_{w(\ell ,j)}}. \end{array}$$

## Appendix D. Isotype parsimony

Here we describe an experimental ranking criterion for GCtree that makes use of observed B-cell isotype data. Antibodies produced by B cells can be separated into a set of isotype classes, according to their heavy-chain constant (C) region. Each individual B-cell expresses a specific C gene, determining the isotype class of its antibodies. As a B-cell line proliferates and undergoes affinity maturation, an irreversible process called class switch recombination takes place. Through this process, B cells in a lineage advance through a fixed sequence of isotype classes [19].

Thus, reconstructed B-cell lineages should obey the isotype class switching order. Using this intuition, data about isotype classes of observed B cells can be informative to phylogenetic inference. Histories that explain the isotype classes of observed genotypes with fewer inferred isotype switching events may be more plausible. We use a modified Fitch–Sankoff algorithm with infinite cost for prohibited transitions and unit cost for all other transitions to reconstruct isotype classes of inferred ancestral nodes in each parsimony tree. The isotype parsimony of a tree is then the number of isotype switching events required along the tree.

This isotype parsimony score may be calculated directly in the history sDAG using the framework described in appendix A. To do so, suppose individuals in the reconstructed tree are expected to express isotype classes in a set I, linearly ordered according to the allowed class switching order. Now define a pseudo-distance function on I, giving the cost of a transition from isotype x to isotype y:

$$\begin{array}{r} d_{I}(x,y)=\left\{ \begin{array}{ll} 0 & x=y\\ \infty & x>y\\ 1 & x<y. \end{array}\right. \end{array}$$

Since any sampled sequence may have been observed in more than one individual (providing the abundance information leveraged by GCtree), each observed (leaf) sequence may correspond to multiple observed isotypes. Taking X to be the set of leaf sequences, let a:X→P(I) express the set of observed isotypes corresponding to each observed sequence.

Consider an internal node v, and a history t in which this node takes part. Regardless of the structure of t, the labels in CU⁡(v) are exactly the labels of the leaf nodes reachable from v. An assignment of inferred ancestral isotypes to internal nodes of t that minimizes isotype switching cost with respect to $d_{I}$ must then assign to v the earliest isotype in the switching order observed among leaf sequences in CU⁡(v). That is, we can define a function H:V→P(I) from nodes in a history sDAG with leaves labelled by X, to sets of isotypes

$$\begin{array}{r} H(\ell ,U)=\left\{ \begin{array}{ll} a(\ell ) & U=\emptyset \\ \left\{\min\left\{x\in a(\ell^{\prime })|\ell^{\prime }\in \mathrm{CU}(\ell ,U)\right\}\right\} & \text{otherwise,} \end{array}\right. \end{array}$$

which assigns to each leaf node the set of corresponding observed isotypes a(ℓ), and to each internal node the singleton set containing the unique optimal inferred isotype for that node.

Finally, we can define the history sDAG edge weight function, which, for an edge $\left(v_{p},v_{c}\right)$ and x the unique element of $H(v_{p})$, takes the value

$$\begin{array}{c} f_{I}(v_{p},v_{c})=\sum_{y\in H(v_{c})}d_{I}(x,y). \end{array}$$

Notice first that $H(v_{c})$ may only contain more than one element if $v_{c}$ is a leaf node. Therefore, we can think of $f_{I}(v_{p},v_{c})$ as a sum of isotype transitions required to explain the observed or inferred isotypes along an edge. This edge-weight function is integer valued. In particular, notice by the choice of H that for any edge $(v_{p},v_{c})\in E$, we know that $\mathrm{CU}(v_{p})⊃\mathrm{CU}(v_{c})$, so for any $x\in H(v_{p}),y\in H(v_{c})$, x≤y and $f_{I}(v_{p},v_{c})\neq \infty$.

A similar, but much more sophisticated tree ranking criterion based on isotype data is provided by the tree inference package TRIBAL [20]. That approach allows isotype data to inform the inferred tree topology directly, rather than using it only to choose between trees inferred using sequence parsimony. In this article, we do not assess the relationship between isotype parsimony and tree correctness. We provide isotype parsimony as an option for users of GCtree, but we emphasize that optimal isotype-informed tree inference should allow isotype data to influence the tree topology search.
